# Integration of the Tumor Mutational Burden and Tumor Heterogeneity Identify an Immunological Subtype of Melanoma With Favorable Survival

**DOI:** 10.3389/fonc.2020.571545

**Published:** 2020-10-30

**Authors:** Yanmei Gao, Chunhe Yang, Ning He, Guodong Zhao, Jianfei Wang, Yadong Yang

**Affiliations:** ^1^ Department of Radiotherapy, Tumor Hospital of Shaanxi Province, Affiliated to the Medical College of Xi’an Jiaotong University, Xi’an, China; ^2^ GloriousMed Clinical Laboratory (Shanghai) Co., Ltd, Shanghai, China

**Keywords:** tumor mutational burden, tumor heterogeneity, immune checkpoint inhibition, melanoma, benefit score

## Abstract

The tumor mutational burden (TMB) has been reported as a predictive marker of the response to immune checkpoint inhibition (ICI) therapy in previous melanoma clinical trials. However, the TMB alone is not sufficient to accurately predict immunotherapy benefit. Additional biomarkers are needed for better stratification of immunotherapy-sensitive patients. In the present study, mutation data and survival information of patients with melanoma were collected from several immunotherapy studies, and tumor heterogeneity was estimated using mutant-allele tumor heterogeneity (MATH). The benefit score was defined as the ratio between the TMB and tumor heterogeneity, and optimal critical values were selected to group patients and evaluate their response to ICI treatment. The benefit score significantly improved the performance of stratifying the overall survival of patients compared with the TMB alone as a predictor in two independent cohorts (p = 0.0068 *vs*. p = 0.1 and p = 0.045 *vs*. p = 0.13), in which patients were treated with Ipilimumab and Nivolumab, respectively. In another cohort of patients with melanoma receiving mixed ICI treatment, the benefit score was also positively associated with higher overall survival (p = 0.022) and outperformed the TMB alone, with a significance of p = 0.089. The benefit score showed a positive correlation with clonal TMB, a reported immunotherapy marker, and exceeded it in immunotherapy response prediction. Besides, a high benefit score was found to be associated with higher proportions of natural killer cells, lower proportions of M2 macrophages and elevated CD8 T cells, all of which favor ICI therapy. In summary, tumor heterogeneity combined with the TMB showed superior efficacy in predicting the response to ICI therapy. This might further help to delineate the mechanisms of immunotherapy in patients with melanoma.

## Introduction

In recent years, immune checkpoint inhibition (ICI) therapy has benefited patients with advanced cancers, as exemplified for lung cancer (1) and melanoma (2). The identification of predictive markers and the mechanisms of resistance to immunotherapy have been subjects of intense research, because only a minority of patients are responsive to checkpoint blockers. Several markers have been proposed, including tumor-infiltrating lymphocytes (3), programmed cell death protein 1 (PD-1) and PD-1 ligand (PD-L1) expression (4), or mutational load (5). A difference in intratumoral PD-L1 expression was observed to contribute to discrepancies in ICI therapy trials and was approved by the FDA as a companion diagnostic for clinical use (6). The tumor mutational burden (TMB) was reported recently as a predictor of pan-cancer survival (7). However, none of these markers has been fully validated to perfectly predict the response to ICI (8, 9).

Among all identified markers, the TMB has proven its potential to predict the response of ICI in melanoma, based on the hypothesis that an elevated TMB corresponds to more neoantigens (10–12). However, it was also found that the effectiveness of the TMB was confounded by disease subtype, whereas a selective response was noticed between different genomic features (13). Putative biomarkers, such as tumor heterogeneity, have been reported to contribute to this variation in the immune response (14). Ultraviolet beta (UVB)-induced heterogeneity was confirmed to be able to diminish the immune response in melanoma, which contributes to loss of efficacy of checkpoint blockade (15), suggesting the potential of tumor heterogeneity as a candidate marker to achieve better performance in ICI response prediction, together with the TMB (16).

In the present study, the TMB and tumor heterogeneity were estimated using somatic mutation data collected from several studies, in which patients with melanoma were treated with checkpoint inhibitors. Next, we defined the benefit score as a metric to integrate the TMB and tumor heterogeneity, and evaluated its predictive performance for the response to ICI therapy.

## Materials and Methods

### Data Collection

After excluding studies without sufficient data, three melanoma cohorts that employed ICI therapy were brought into the analysis: The Van Allen cohort (12), the Miao cohort (17), and the Riaz cohort (18). Genomic profiling based on whole exome sequencing (WES) and clinical characteristics, such as age, sex, tumor stage, and survival information were gathered from publicly accessible resources. Somatic mutations and copy number segmentation data of 206 patients with melanoma profiled by The Cancer Genome Atlas (TCGA) were obtained from a previous pan-cancer report (19).

### The Benefit Score

We defined the benefit score as the ratio between the TMB and tumor heterogeneity, where the TMB denoted the number of nonsynonymous somatic mutations and heterogeneity was determined using mutant-allele tumor heterogeneity (MATH), as calculated using the R package, maftools (20). For the convenience of calculation, MATH was set to 1 when its value was equal to zero. Mutations with allelic fractions less than 0.05 or a coverage of no more than 30 were excluded, as described previously (12).

### Clonal Tumor Mutation Load

The clonal TMB, representing the number of clonal nonsynonymous somatic mutations, is a measure used to evaluate the clonal tumor mutation load of patients. In the Riaz cohort, cancer cell fraction (CCF) of mutations was estimated using PyClone (21), and mutations with a lower confidential interval of CCF exceeded 95% were defined as clonal mutations. In the Miao cohort, mutational clonality was assessed using ABSOLUTE (22), and mutations with a CCF of 1, or those whose probability of being clonal exceeded the probability of being subclonal, were identified clonal. Clonal mutations in the TCGA dataset were determined using the same method as in the Miao cohort.

### Statistical Analysis

Correlation analysis between the TMB and MATH, as well as the benefit score and the clonal TMB, was implemented using the Spearman method. Patients in each cohort were stratified into two groups based on the critical value of the benefit score inferred using the function surv_cutpoint in the R package survival, using the maximally selected log-rank statistic; the log-rank test was also performed. The prognostic significance of the benefit score was examined using a Cox multivariate regression model that incorporated clinical and molecular features, and the hazard ratios (HRs) were calculated. Fraction data of each tumor immune cell type in the Riaz cohort were downloaded from The Cancer Imaging Archive (TCIA) (https://tcia.at/home) (8). Cell fractions before treatment and fraction changes during treatment were compared between the high and low benefit score sets using a two-tailed Wilcoxon test.

## Results

### The Benefit Score as a Metric for Integration of the TMB and Tumor Heterogeneity

**Figure 1:**
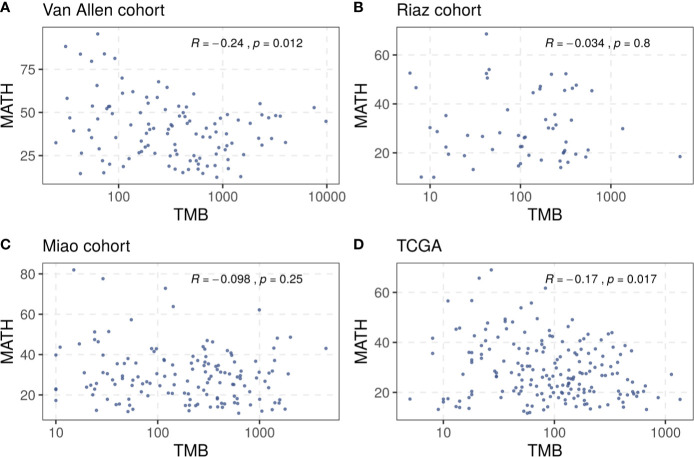
Correlation between TMB and tumor heterogeneity in **(A)** the Van Allen cohort, **(B)** the Riaz cohort, **(C)** the Miao cohort, and **(D)** the TCGA dataset. Spearman correlation coefficients and statistical significance are labeled at the top right in each part.

A total of 321 patients with melanoma from three cohorts, the Van Allen cohort, the Riaz cohort, and the Miao cohort, were included in our investigation (**Supplementary Table S1**). The TMB and tumor heterogeneity were measured independently using the data collected from each cohort. A small number of samples, four in the Riaz cohort and nine in the Miao cohort, were excluded from subsequent analysis because the tumor heterogeneity could not be estimated due to an inadequate number of mutations. The TMB did not correlate clearly with the MATH value in these cohorts and a similar result was observed in the TCGA melanoma dataset (**Figure 1**), rationalizing the integration of these two factors to merge their prediction effects. Consequently, we defined the benefit score as the TMB divided by the MATH value as a proxy for tumor heterogeneity.

### The Association Between the Benefit Score and the Clonal TMB

**Figure 2:**
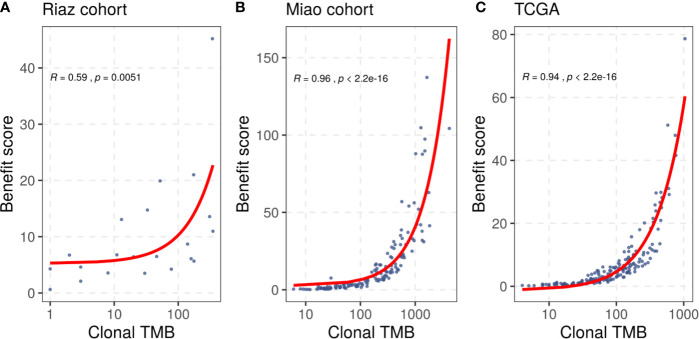
Correlation between the benefit score and clonal TMB in **(A)** the Riaz cohort, **(B)** the Miao cohort, and **(C)** the TCGA dataset. Spearman correlation coefficients and statistical significance are labeled at the top left in each part.

In addition to the benefit score, the clonal TMB is also simultaneously related to the TMB and tumor heterogeneity, and was reported to be associated with the response of patients treated with checkpoint blockers. We compared the benefit score with the clonal TMB based on the clonal results collected from the included studies and the correlation result is shown in **Figure 2**. We found that the benefit score correlated positively with the clonal TMB in the Riaz cohort (R = 0.59, p = 0.0051) and the Miao cohort (R = 0.96, p < 0.001). A similar trend was observed in the TCGA melanoma dataset (R = 0.94, p < 0.001). The diversity in the significance of the correlation between the Riaz cohort and the other two cohorts might have been caused by the number of patients and approach used to estimate clonality.

### The Benefit Score Yields a Better ICI Response Prediction Performance

**Figure 3:**
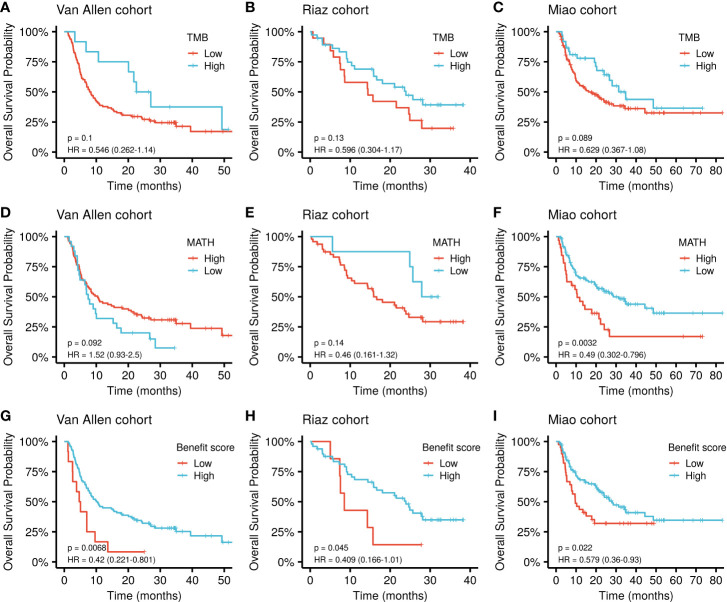
Kaplan-Meier curve of overall survival by TMB, MATH, and benefit scores in different datasets. **(A–C)** Survival curves for the TMB in each cohort. **(D–F**) Survival curves for MATH in each cohort. **(G–I)** Survival curves for the benefit score in each cohort. P values of the log-rank test are shown at the top right for each curve.

The cut-off value for each marker was determined based on the principle of comparing their best performance to stratify the patients (**Supplementary Table S2**). The benefit score showed a significantly improved performance in stratifying the overall survival (OS) of patients receiving ICI treatment compared with that of the TMB alone as a predictor in the Van Allen cohort (p = 0.0068, HR = 0.42 *vs*. p = 0.1, HR = 0.546; **Figure 3A**). The same results were obtained using the Riaz cohort (p = 0.045, HR = 0.409 *vs*. p = 0.13, HR = 0.596; **Figure 3B**) and the Miao cohort (p = 0.022, HR = 0.579 *vs.* p = 0.089, HR = 0.629; **Figure 3C**). Meanwhile, the benefit score also exceeded the ability of MATH to predicting higher OS in the Van Allen cohort (p = 0.0068, HR = 0.42 *vs*. p = 0.092, HR = 1.52; **Figure 3D**) and the Riaz cohort (p = 0.045, HR = 0.409 vs. p = 0.14, HR = 0.46; **Figure 3E**). Only in the Miao cohort, MATH was superior to the benefit score (p = 0.0032, HR = 0.49 vs. p = 0.022, HR = 0.579; **Figure 3F**). Survival analysis result of the benefit score was is shown in **Figures 3G–I**. In addition, the superiority of the benefit score over the clonal TMB was demonstrated in the Riaz cohort (p = 0.045, HR = 0.409 *v*s. p = 0.33, HR = 0.664; **Supplementary Figure S1A**) and the Miao cohort (p = 0.022, HR = 0.579 *vs*. p = 0.037, HR = 0.624; **Supplementary Figure S1B**). A slight difference, without statistical significance, was observed when simultaneously considering the clonal TMB and the benefit score, indicating synergistic effects might exist between these two features (**Supplementary Figures S1C, D**).

Cox multivariate regression was implemented to verify the benefit score’s prediction of ICI therapy response, independently of other clinical or molecular factors. Relevant covariates including age, sex, tumor stage, previous treatment, and driver gene mutations, together with the benefit score, were incorporated into the model. The result demonstrated that a low benefit score was independently associated with shorter OS, after adjusting for age, sex, and tumor stage in the Van Allen cohort (HR = 2.16, p = 0.02, **Table 1**). Despite that fact that a similar result was not significant in the other two cohorts, a lower benefit score still had a higher hazard ratio when correcting for age and sex in the Miao cohort (HR = 1.30, p = 0.308, **Table 1**), and while controlling for *BRAF*, *NF1*, and *RAS* mutations, and previous Ipilimumab treatment, in the Riaz cohort (HR = 2.31, p = 0.085, **Table 1**). The benefit score outperformed the TMB, with more significantly higher hazard ratios, as estimated by multivariate regression, in all cohorts (**Supplementary Table S3**).

**Table 1:** Multivariate Cox regression analysis of overall survival with the benefit score and other covariates in different cohorts.

Cohort	Covariates	HR	95% CI	P-value
Van Allen cohort	Age	1.00	0.99–1.02	0.629
	Sex			
	Male	0.73	0.45–1.18	0.200
	Stage			
	IV	4.20	1.31–13.5	0.016^*^
	Benefit score			
	Low	2.16	1.13–4.12	0.020^*^
Riaz cohort	Mutation Subtype			
	*BRAF*	0.90	0.40–2.04	0.802
	*NF1*	1.16	0.32–4.16	0.822
	*RAS*	0.68	0.27–1.73	0.420
	Prior treatment			
	Yes	0.89	0.44–1.78	0.738
	Benefit score			
	Low	2.31	0.89–6.01	0.085
Miao cohort	Age	1.01	1.00–1.03	0.135
	Sex			
	Male	0.77	0.45–1.32	0.344
	Benefit score			
	Low	1.30	0.79–2.14	0.308

HR, Hazard Ratio; CI, Confidence Interval; *indicates statistical significance.

### The Benefit Score and the Tumor Microenvironment

**Figure 4:**
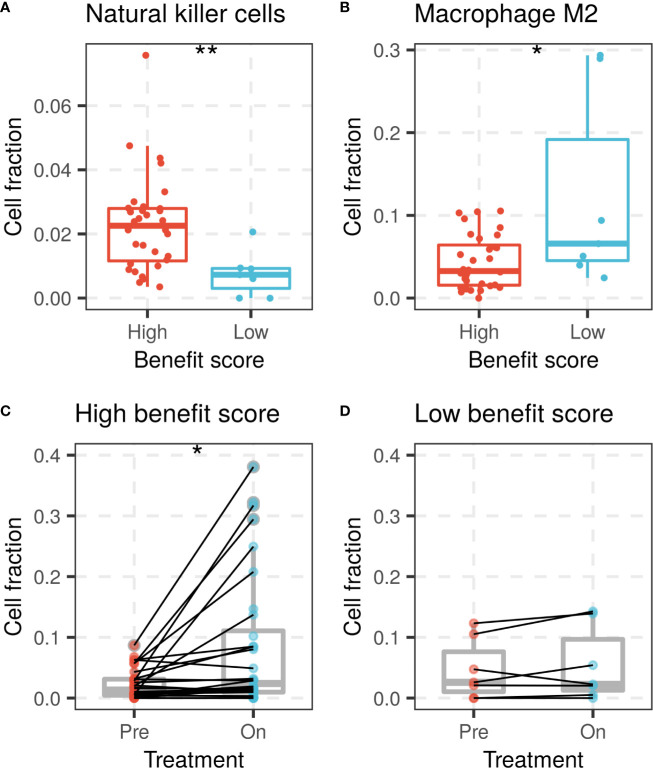
NK cells and macrophage M2 fractions of patients between high and low benefit score groups in the Riaz cohort. **(A)** NK cell fraction of patients grouped by benefit score is shown by a box plot at left, while **(B)** the macrophage M2 fraction is shown on the right side. CD8 T cell fraction change during ICI treatment in **(C)** high benefit score group and **(D)** low benefit score group. (*p < 0.05, **p < 0.01).

The benefit score was also investigated for its association with variation in the tumor microenvironment, mainly the immune cell composition. Immune cell proportions were calculated from gene expression profiles and compared between the high and low benefit score groups in the Riaz cohort. A high benefit score tumor was significantly associated with a higher proportion of activated natural killer (NK) cells and a lower proportion of M2 macrophages compared with those in the low benefit score group (p = 0.0041 and p = 0.049, respectively, **Figures 4A, B**). A significant elevation of the CD8 T cell fraction during treatment was found in the high benefit score group, but not in the low benefit score group (p = 0.014 and p = 0.29, respectively, **Figures 4C, D**). The observation that higher proportions of NK cells, lower proportions of M2 macrophages and elevated CD8 T cells are favorable factors for immunotherapy supports the result that the benefit score is a remarkable predictor.

## Discussion

The ability of the TMB to predict the response to immunotherapy response has been reported to be slightly restrictive in recent research (13, 23). In the present study, we combined the TMB with tumor heterogeneity to form the benefit score as a new indicator to achieve a better predictive effect compared with that of the TMB alone. The observed prognostic significance was consistent with previous reports, proving that integration of the TMB and tumor heterogeneity forms a superior predictive marker for the response to ICI treatment.

A high TMB is a relative measure of a tumor being more immunogenic by producing more neoantigens. However, the effectiveness of the antitumor immune response would be greatly reduced if the presented antigens were subclone-specific, because immune surveillance is promoted by clonal neoantigens (14). The chance of a neoantigen being clonal cannot be reflected by the TMB and high neoantigen heterogeneity usually originates from a more complicated tumor evolutionary structure. Our benefit score covers both the neoantigen burden and clonality, without direct clonal neoantigen measurement. A high benefit score corresponds to either a high mutational load, low genetic heterogeneity, or both, i.e., it reflects a high neoantigen load and the avoidance of immune evasion mediated by tumor heterogeneity, which would likely be more sensitive to immunotherapy. Similar to our benefit score, the clonal TMB considers clonality and the neoantigen burden simultaneously. It also strongly correlates with the benefit score, except that the clonal TMB showed a poorer performance than the benefit score to predict the ICI response, because the immune response induced by tumor-specific epitopes generated from subclones are neglected by the clonal TMB.

It has been reported that NK cells contribute to PD-1/PD-L1 antibody-mediated immunotherapy by expressing PD-L1 as a cytolytic effector (24, 25), while M2 macrophages help to generate an immune-evasive microenvironment in evolved cancers (26, 27). A high benefit score was found to be associated with a higher proportion of NK cells, a lower proportion of M2 macrophages and elevated CD8 T cells in the Riaz cohort, in which patients were treated with Nivolumab, in accordance with its favorable survival prediction.

Despite of significant results achieved in the present study, a disadvantage common in most cases involving heterogeneity evaluation was that genetic diversity might not be adequately estimated using *in silico* methods in a single tumor region sample, which could lead to unstable results. Such a limitation, together with the small cohort, might have interfered with the robustness of the multivariate analysis, resulting in an insignificant outcome. Analysis of multiple regions or single cell sequencing in a larger cohort would be good methods to validate the prognostic value of our findings in the future.

In summary, our study suggested that integration of the tumor mutational load and heterogeneity provides a better predictive marker of the response to ICI treatment for patients with melanoma. The potential significance of the benefit score could be further investigated and applied using more sophisticated approaches in other types of cancer.

## Data Availability Statement

The original contributions presented in the study are included in the article/**Supplementary Material**. Further inquiries can be directed to the corresponding authors.

## Author Contributions

YG, CY, YY, and GZ conceived and designed the study. YG, CY, NH, YY, and JW conducted data collection, analysis, and interpretation. YG and CY wrote the manuscript. All authors contributed to the article and approved the submitted version.

## Conflict of Interest

Authors CY, NH, GZ, JW, and YY were employed by the company GloriousMed Clinical Laboratory (Shanghai) Co., Ltd.

The remaining author declares that the research was conducted in the absence of any commercial or financial relationships that could be construed as a potential conflict of interest.

The handling editor declared a past co-authorship with one of the authors YY.

## References

[1] Borghaei H Paz-Ares L Horn L Spigel DR Steins M Ready NE Nivolumab versus Docetaxel in Advanced Nonsquamous Non-Small-Cell Lung Cancer. N Engl J Med (2015) 373(17):1627–39. 10.1056/NEJMoa1507643 PMC5705936 26412456

[2] Hodi FS O’Day SJ McDermott DF Weber RW Sosman JA Haanen JB Improved survival with ipilimumab in patients with metastatic melanoma. N Engl J Med (2010) 363(8):711–23. 10.1056/NEJMoa1003466 PMC3549297 20525992

[3] Tang H Wang Y Chlewicki LK Zhang Y Guo J Liang W Facilitating T Cell Infiltration in Tumor Microenvironment Overcomes Resistance to PD-L1 Blockade. Cancer Cell (2016) 30(3):500. 10.1016/j.ccell.2016.08.011 27622338

[4] Patel SP Kurzrock R PD-L1 Expression as a Predictive Biomarker in Cancer Immunotherapy. Mol Cancer Ther (2015) 14(4):847–56. 10.1158/1535-7163.MCT-14-0983 25695955

[5] Rizvi NA Hellmann MD Snyder A Kvistborg P Makarov V Havel JJ Mutational landscape determines sensitivity to PD-1 blockade in non-small cell lung cancer. Science (2015) 348(6230):124–8. 10.1126/science.aaa1348 PMC4993154 25765070

[6] Garon EB Rizvi NA Hui R Leighl N Balmanoukian AS Eder JP Pembrolizumab for the Treatment of Non–Small-Cell Lung Cancer. N Engl J Med (2015) 372(21):2018–28. 10.1056/NEJMoa1501824 25891174

[7] Samstein RM Lee CH Shoushtari AN Hellmann MD Shen R Janjigian YY Tumor mutational load predicts survival after immunotherapy across multiple cancer types. Nat Genet (2019) 51(2):202–6. 10.1038/s41588-018-0312-8 PMC6365097 30643254

[8] Charoentong P Finotello F Angelova M Mayer C Efremova M Rieder D Pan-cancer Immunogenomic Analyses Reveal Genotype-Immunophenotype Relationships and Predictors of Response to Checkpoint Blockade. Cell Rep (2017) 18(1):248–62. 10.1016/j.celrep.2016.12.019 28052254

[9] Spencer KR Wang J Silk AW Ganesan S Kaufman HL Mehnert JM Biomarkers for Immunotherapy: Current Developments and Challenges. Am Soc Clin Oncol Educ Book (2016) 35:e493–503. 10.1200/EDBK_160766 27249758

[10] Eroglu Z Zaretsky JM Hu-Lieskovan S Kim DW Algazi A Johnson DB High response rate to PD-1 blockade in desmoplastic melanomas. Nature (2018) 553(7688):347–50. 10.1038/nature25187 PMC5773412 29320474

[11] Snyder A Makarov V Merghoub T Yuan J Zaretsky JM Desrichard A Genetic basis for clinical response to CTLA-4 blockade in melanoma. N Engl J Med (2014) 371(23):2189–99. 10.1056/NEJMoa1406498 PMC4315319 25409260

[12] Van Allen EM Miao D Schilling B Shukla SA Blank C Zimmer L Genomic correlates of response to CTLA-4 blockade in metastatic melanoma. Science (2015) 350(6257):207–11. 10.1126/science.aad0095 PMC5054517 26359337

[13] Liu D Schilling B Liu D Sucker A Livingstone E Jerby-Amon L Integrative molecular and clinical modeling of clinical outcomes to PD1 blockade in patients with metastatic melanoma. Nat Med (2019) 25(12):1916–27. 10.1038/s41591-019-0654-5 PMC6898788 31792460

[14] McGranahan N Furness AJ Rosenthal R Ramskov S Lyngaa R Saini SK Clonal neoantigens elicit T cell immunoreactivity and sensitivity to immune checkpoint blockade. Science (2016) 351(6280):1463–9. 10.1126/science.aaf1490 PMC4984254 26940869

[15] Wolf Y Bartok O Patkar S Eli GB Cohen S Litchfield K UVB-Induced Tumor Heterogeneity Diminishes Immune Response in Melanoma. Cell (2019) 179(1):219–35 e21. 10.1016/j.cell.2019.08.032 31522890 PMC6863386

[16] Kim K Kim HS Kim JY Jung H Sun JM Ahn JS Predicting clinical benefit of immunotherapy by antigenic or functional mutations affecting tumour immunogenicity. Nat Commun (2020) 11(1):951. 10.1038/s41467-020-14562-z 32075964 PMC7031381

[17] Miao D Margolis CA Vokes NI Liu D Taylor-Weiner A Wankowicz SM Genomic correlates of response to immune checkpoint blockade in microsatellite-stable solid tumors. Nat Genet (2018) 50(9):1271–81. 10.1038/s41588-018-0200-2 PMC6119118 30150660

[18] Riaz N Havel JJ Makarov V Desrichard A Urba WJ Sims JS Tumor and Microenvironment Evolution during Immunotherapy with Nivolumab. Cell (2017) 171: (4):934–49 e16. 10.1016/j.cell.2017.09.028 29033130 PMC5685550

[19] Raynaud F Mina M Tavernari D Ciriello G Pan-cancer inference of intra-tumor heterogeneity reveals associations with different forms of genomic instability. PLoS Genet (2018) 14(9):e1007669. 10.1371/journal.pgen.1007669 30212491 PMC6155543

[20] Mayakonda A Lin DC Assenov Y Plass C Koeffler HP Maftools: efficient and comprehensive analysis of somatic variants in cancer. Genome Res (2018) 28(11):1747–56. 10.1101/gr.239244.118 PMC6211645 30341162

[21] Roth A Khattra J Yap D Wan A Laks E Biele J PyClone: statistical inference of clonal population structure in cancer. Nat Methods (2014) 11(4):396–8. 10.1038/nmeth.2883 PMC4864026 24633410

[22] Carter SL Cibulskis K Helman E McKenna A Shen H Zack T Absolute quantification of somatic DNA alterations in human cancer. Nat Biotechnol (2012) 30(5):413–21. 10.1038/nbt.2203 PMC4383288 22544022

[23] Hellmann MD Ciuleanu TE Pluzanski A Lee JS Otterson GA Audigier-Valette C Nivolumab plus Ipilimumab in Lung Cancer with a High Tumor Mutational Burden. N Engl J Med (2018) 378(22):2093–104. 10.1056/NEJMoa1801946 PMC7193684 29658845

[24] Hsu J Hodgins JJ Marathe M Nicolai CJ Bourgeois-Daigneault MC Trevino TN Contribution of NK cells to immunotherapy mediated by PD-1/PD-L1 blockade. J Clin Invest (2018) 128(10):4654–68. 10.1172/JCI99317 PMC6159991 30198904

[25] Dong W Wu X Ma S Wang Y Nalin AP Zhu Z The Mechanism of Anti-PD-L1 Antibody Efficacy against PD-L1-Negative Tumors Identifies NK Cells Expressing PD-L1 as a Cytolytic Effector. Cancer Discov (2019) 9(10):1422–37. 10.1158/2159-8290.CD-18-1259 PMC7253691 31340937

[26] Qian BZ Pollard JW Macrophage diversity enhances tumor progression and metastasis. Cell (2010) 141(1):39–51. 10.1016/j.cell.2010.03.014 20371344 PMC4994190

[27] Mantovani A Marchesi F Malesci A Laghi L Allavena P Tumour-associated macrophages as treatment targets in oncology. Nat Rev Clin Oncol (2017) 14(7):399–416. 10.1038/nrclinonc.2016.217 28117416 PMC5480600

