# Supplementary material for: Integration of the Tumor Mutational Burden and Tumor Heterogeneity Identify an Immunological Subtype of Melanoma With Favorable Survival
Source: Front Oncol. 2020 Oct 30;10:571545. doi: 10.3389/fonc.2020.571545 (PMC7661856; doi:10.3389/fonc.2020.571545)
Supplement: Supplementary file 1 [file Table_1.docx]

Supplementary Material


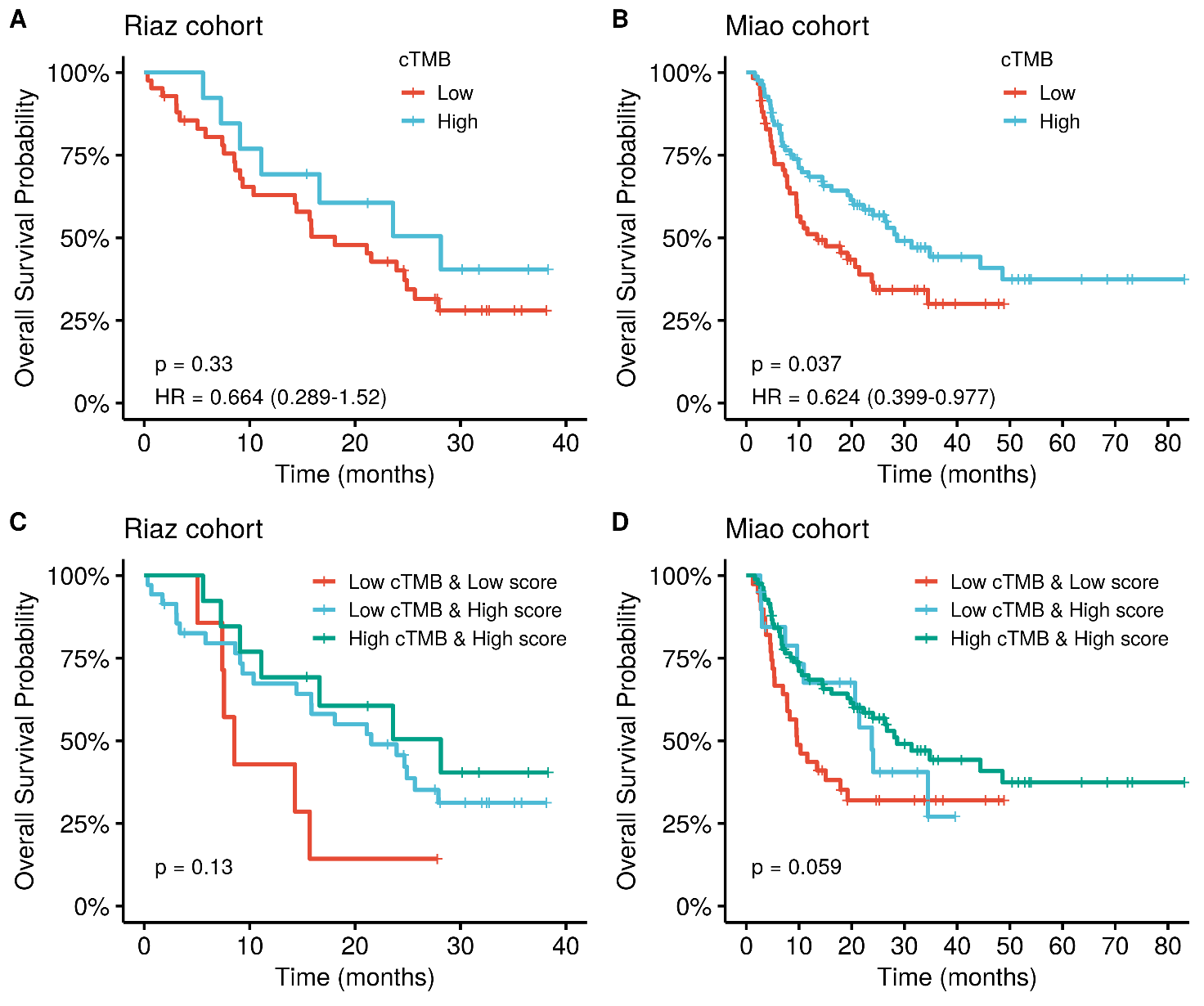


**Supplementary Figure 1.** Kaplan-Meier curve of overall survival by clonal TMB in (A) Riaz cohort and (B) Miao cohort and Kaplan-Meier curve of overall survival by clonal TMB & benefit score in (C) Riaz cohort and (D) Miao cohort. P value of log-rank test and hazard ratio with 95% confidence interval are shown at bottom left for each curve.

**Supplementary Table 1.** Characteristics of included melanoma cohorts

| Characteristics | Van Allen Cohort  (N=110) | Riaz cohort  (N=56) | Miao cohort  (N=142) |
| --- | --- | --- | --- |
| Age, median (range) | 61.5 (18-86) | - | 63 (18-86) |
| Gender, male (percentage) | 78 (71.9%) | - | 93 (65.5%) |
| Treatment | Ipilimumab | Nivolumab | Mixed treatment |
| OS, median months (range) | 9.05 (1.13-54.4) | 17.4 (0.33-38.3) | 15.6 (1.22-83.1) |

Van Allen cohort data source: https://science.sciencemag.org/content/suppl/2015/09/09/science.aad0095.DC1

Riaz cohort data source: https://github.com/riazn/bms038_analysis

Miao cohort data source: https://www.cbioportal.org/study/summary?id=mixed_allen_2018

**Supplementary Table 2.** Cut-offs of TMB, MATH and benefit score for stratifying patients in different cohorts

| Cohort | Cut-off | | |
| --- | --- | --- | --- |
|  | TMB | MATH | Benefit score |
| Van Allen cohort | 1587 | 25.54 | 1.13 |
| Riaz cohort | 59 | 16.67 | 0.67 |
| Miao cohort | 524 | 37.12 | 2.26 |

**Supplementary Table 3.** Multivariable cox regression of overall survival with TMB, MATH & benefit score in different cohorts

| Cohort | Covariates | HR | 95% CI | P-value |
| --- | --- | --- | --- | --- |
| Van Allen cohort | TMB |  |  |  |
|  | Low | 2.12 | 1.01-4.45 | 0.047^*^ |
|  | MATH |  |  |  |
|  | High | 0.68 | 0.41-1.11 | 0.123 |
|  | Benefit score |  |  |  |
|  | Low | 2.16 | 1.13-4.12 | 0.020^*^ |
| Riaz cohort | TMB |  |  |  |
|  | Low | 1.61 | 0.78-3.34 | 0.197 |
|  | MATH |  |  |  |
|  | High | 2.15 | 0.74-6.22 | 0.157 |
|  | Benefit score |  |  |  |
|  | Low | 2.31 | 0.89-6.01 | 0.085 |
| Miao cohort | TMB |  |  |  |
|  | Low | 1.08 | 0.59-2.00 | 0.796 |
|  | MATH |  |  |  |
|  | High | 2.04 | 1.20-3.47 | 0.009* |
|  | Benefit score |  |  |  |
|  | Low | 1.30 | 0.79-2.14 | 0.308 |

HR = Hazard Ratio, CI = Confidence Interval
